# 肿瘤微环境内的免疫细胞、PD-1与EGFR-TKIs疗效关系新进展

**DOI:** 10.3779/j.issn.1009-3419.2017.11.09

**Published:** 2017-11-20

**Authors:** 世洋 袁, 慧 徐, 军平 谢

**Affiliations:** 330006 南昌，南昌大学第二附属医院呼吸与危重症医学科 Department of Respiratory and Critical Care Medicine, the Second Affiliated Hospital of Nanchang University, Nanchang 330006, China

**Keywords:** 肿瘤微环境, 肺肿瘤, 靶向治疗, 免疫细胞, EGFR-TKIs, PD-1, Tumor microenvironment, Lung neoplasms, Targeted therapy, Immune cells, EGFR-TKIs, Programed cell death protein 1

## Abstract

表皮生长因子受体酪氨酸激酶抑制剂（epidermal growth factor receptor-tyrosine kinase inhibitors, EGFR-TKIs）靶向治疗如今成为EGFR基因突变的晚期非小细胞肺癌（non-small cell lung cancer, NSCLC）患者的主导治疗方式，但随着用药时间的延长，大部分患者出现靶向药物的耐药。肿瘤微环境是肿瘤细胞赖以生存和发展的内环境，微环境中调节T（regulatory T, Treg）细胞、树突状细胞、巨噬细胞、成纤维细胞等免疫细胞介导的免疫反应以及程序性死亡受体1（programed cell death protein 1, PD-1）及其配体PD-1L/PD-2L可能参与EGFR-TKIs的耐药形成，现本综述将阐述肿瘤微环境中免疫细胞对EGFR-TKIs靶向治疗疗效相互影响的可能机制，以期寻求新的靶点，进一步提高EGFR-TKIs的抗肿瘤疗效和延长有效时间。

## Abstract (en)

In recent years, targeted therapy of epidermal growth factor receptor-tyrosine kinase inhibitor (EGFR-TKIs) is the leading treatment modality for patients with advanced non-small cell lung cancer (NSCLC) and *EGFR* gene mutation. However, with the prolongation of the medication time, most of the patients appeared drug resistance. Tumor microenvironment is the internal environment for the survival and development of tumor cells. The immune response which mediated by immune cells, like regulatory T (Treg), dendritic cells, macrophages, fibroblasts, *etc*. And the programmed cell death receptor 1 (PD-1) with its ligand PD-1L/PD-2L may participate in the drug resistance of EGFR-TKIs. This review will elaborate the possible mechanism of the interaction of immune cells on EGFR-TKIs in the tumor microenvironment, in order to seek new targets, and further improve the anti-tumor efficacy and prolong the effective time of EGFR-TKIs.

分子靶向药物治疗是晚期肿瘤治疗的研究热点之一，因其良好的疗效和可耐受的毒副作用而越来越被人们所接受。其中，表皮生长因子受体酪氨酸激酶抑制剂（epidermal growth factor receptor tyrosine-kinase inhibitors, EGFR-TKIs）在晚期非小细胞肺癌（non-small cell lung cancer, NSCLC）治疗方面已取得了突破性进展，其代表药物有吉非替尼和厄洛替尼等，目前为*EGFR*基因敏感突变并且不存在耐药基因的晚期NSCLC患者推荐EGFR-TKIs一线治疗得到全世界范围内的认可^[1-3]^。但是，随着用药时间的延长（中位时间为9个月-13个月），大部分患者对EGFR-TKIs产生耐药^[4-6]^，对EGFR-TKIs耐药机制及如何逆转耐药的研究也成为国内外研究的热点。肿瘤微环境是肿瘤细胞赖以生存和发展的内环境，近年来肿瘤微环境中的免疫细胞和其他细胞或因子的相互作用逐渐被人们所认识^[7-9]^，肿瘤内微环境内的免疫反应与EGFR-TKIs抗肿瘤反应之间会产生相互影响^[10]^。本综述将阐述肿瘤微环境中免疫细胞、PD-1对EGFR-TKIs靶向治疗疗效相互影响的可能机制，以期寻求新的治疗靶点，进一步提高EGFR-TKIs的抗肿瘤疗效和延长有效时间。

## 1 肿瘤微环境内的免疫细胞与EGFR-TKIs疗效的关系

### 1.1 调节性T细胞

CD4^+^ T细胞和CD8^+^ T细胞是公认的抗肿瘤免疫细胞，通过释放*γ*-干扰素、穿孔素和颗粒酶B等肿瘤毒性细胞因子达到杀死肿瘤细胞的作用。Treg细胞是一类控制自身免疫反应的细胞群，肿瘤内的Treg细胞分泌的转化生长因子（transforming growth factor, TGF）-β和白介素（interleukin, IL）-10能产生一个免疫抑制环境，有助于减弱CD4^+^ T细胞、CD8^+^ T细胞及NK细胞产生的抗肿瘤效应^[11]^。实验^[12]^证实，在不同小鼠模型中CD25^+^ Treg的消耗极大地提高了小鼠的抗肿瘤免疫效应。肿瘤患者通常存在抗肿瘤免疫细胞功能失调，在肺癌、乳腺癌、黑色素瘤等肿瘤组织内部和边缘，Treg细胞的数量明显增加^[13-15]^。Huang等^[16]^的研究证实，EGFR^+^外泌体诱导生成肿瘤特异性Treg细胞，下调肿瘤特异性CD8^+^ T细胞的表达，从而影响抗肿瘤疗效。研究^[17]^表明，核内转录因子FoxP3是功能性Treg的特异性标志。Wang等^[18]^研究发现，EGFR信号可以通过抑制糖原合酶激酶-3β（glycogen synthase kinase 3β, GSK-3β）活性来增强FoxP3蛋白的表达，而使用EGFR-TKI吉非替尼可以修复GSK-3β活性和减弱Treg细胞免疫抑制功能。He等^[19]^在肺鳞癌的小鼠模型中，观察到敲除EGFR基因后形成的肿瘤微环境中浸润的FoxP3^+^ Treg细胞较少，而野生型成瘤模型使用吉非替尼治疗1 wk后，同样观察到FoxP3^+^ Treg细胞、FoxP3 RNA表达减少的趋势。其认为，EGFR依赖性Treg细胞增强肺鳞癌的生长，相对短暂的系统性抑制EGFR信号通路可改变靶向肿瘤的免疫微环境。无独有偶，Mascia等^[20]^的动物实验中也得到了类似的研究结果，系统性EGFR抑制剂可以明显抑制肿瘤细胞的生长和微环境中Treg细胞的渗入，EGFR的靶向抗肿瘤治疗可以影响肿瘤细胞自身免疫调节，而Treg可能是EGFR抑制剂抗肿瘤活性的一个靶点。

### 1.2 树突状细胞（dendritic cells, DC）

DC在免疫系统中是最有效的专职抗原提呈细胞。在非成熟状态，充当免疫系统的哨点，持续地巡查机体环境内的抗原；同时，DC拥有把外源性抗原内源化的独特能力，即所谓的交叉提呈，如把肿瘤相关抗原提呈给CD8^+^ T细胞；在成熟状态下，DC迁移至淋巴器官内，把抗原提呈给幼稚T细胞，活化的T细胞增殖分化后离开淋巴结搜寻和杀死抗原依赖的细胞^[21]^。在多项临床实验中证实，DC疫苗是安全可行的，以DC为基础的联合化疗、靶向药物以及免疫检查点抑制剂等制备的疫苗为肿瘤治疗的提供多种思路，而Sipuleucel-T在晚期前列腺癌患者三期临床实验中被证实显著的生存获益^[21]^。Wang等^[22]^的研究探讨了共培养DC和细胞因子诱导的杀伤细胞（cytokine-induced killer cells, CIK）在A549肺癌细胞株的杀伤作用和可能机制，其结果认为DC-CIK可能通过诱导增强IFN-*γ*蛋白表达、促进PGE-2、caspase-3/9和IL-10活性以及抑制IL-4的活性和TGF-β蛋白、EGFR蛋白的表达来抑制A549肺癌细胞的生长和增加细胞毒性发挥抗肿瘤效应，为DC-CIK细胞共培养免疫治疗NSCLC进入临床提供实验基础，同时也为EGFR-TKIs耐药后的治疗提供了新的思路。段琼等^[23]^的研究首先富集培养人EGFR靶向药吉非替尼耐药的肺癌A549、H292干细胞，分离获得耐药肺癌A549、H292干细胞膜微粒；分离培养人DC和CIK细胞，将肺癌干细胞膜微粒负载DC-CIK细胞，检测肺癌干细胞膜微粒负载DC-CIK细胞对耐药肺癌细胞A549、H292的杀伤性；以肺癌干细胞膜微粒负载DC-CIK细胞的分泌上清作用于肺癌干细胞，观察其对肺癌干细胞凋亡的影响，以期为针对EGFR-TKIs耐药肺癌的免疫细胞治疗提供实验基础。其结果表明肺癌干细胞膜微粒负载DC-CIK细胞对于EGFR-TKIs耐药肺癌干细胞具有促进凋亡作用，这为EGFR-TKIs耐药肺癌的临床治疗和防止复发提供了新的治疗途径和实验依据。

### 1.3 肿瘤相关的巨噬细胞（tumor-associated macrophages, TAMs）

TAMs来自于未成熟的单核细胞，被大量肿瘤源性细胞因子（如CSF-1、IL-6、IL-10以及一系列化学趋化因子等），募集到肿瘤区域，TAMs在肿瘤微环境的诱导下发生表型和功能的转换，并再产生趋化因子、生长因子、血管生成因子和基质蛋白酶等化学因子作用于肿瘤细胞，进而促进肿瘤生长、侵袭与转移^[24-26]^。章必成等^[27]^研究不同活化表型的巨噬细胞对Lewis小鼠肺癌的细胞增殖和侵袭的影响，结果表明M1型TAMs可产生NO，使Lewis小鼠肺癌细胞增殖和侵袭受到抑制，而M2型TAMs因为能够高表达血管内皮生长因子（vascular endothelial growth factor, VEGF）-C，所以拥有促进Lewis小鼠肺癌细胞增殖和侵袭能力，且随着肿瘤进展微环境的变化可以使M1型巨噬细胞向M2型巨噬细胞方向极化。M2型TAMs低表达IL-12，高表达IL-10，诱导Ⅱ型免疫应答，其肿瘤杀伤活性低，因子TAMs功能更接近M2型巨噬细胞的功能表型。Chung等^[28]^研究认为TAMs浸润降低EGFR-TKIs一线治疗的敏感性。此外，Zhang等^[29]^的临床研究认为晚期肺腺癌M2-TAMs浸润与EGFR-TKIs治疗疗效呈负相关。但是均未阐明TAMs或M2-TAMs影响EGFR-TKI敏感性的具体机制。有研究^[30]^在索拉非尼耐药的肝癌细胞中加入M2-TAMs后，其生长速率明显高于对照组，索拉非尼耐药的肝癌细胞中的β-catenin、EGFR的蛋白表达均高于对照组，以及获得索拉非尼耐药的肝癌细胞的增殖、侵袭、迁移能力明显增加；其结论认为TAMs和EGFR/β-catenin信号通路促进索拉非尼耐药肝癌细胞的增殖、侵袭和迁移。综上所述，TAMs不仅促进肿瘤的发生和发展，也对EGFR-TKIs的疗效产生影响。因此阻止TAMs的募集或逆转TAMs的极化的有关策略也成为研究热点。我们前期也综述了逆转TAMs极化的有关策略^[31]^，但在EGFR-TKIs耐药的肺癌患者中，有关针对TAMs靶向相关的研究甚少，有待进一步的研究。

### 1.4 肿瘤相关成纤维细胞（cancer-associated ﬁbroblasts, CAFs）

CAFs是一类不同细胞源性的细胞群，是肿瘤微环境的重要组成部分，近年来研究^[32-34]^发现，其通过多种介导机制，影响肿瘤能量代谢，促进血管生成，促进肿瘤的发生、发展。Choe等^[35]^的研究共培养了CAFs和PC9细胞，使PC9对EGFR-TKI厄洛替尼产生了耐药性[IC_50_ < 0.01 µmol/L，转变为IC_50_=0.05 µmol/L（*P* < 0.05）]，其机制可能和hedgehog（Hh）信号通路激活和CAF诱导PC9细胞表型上皮间质转化（epithelial-mesenchymal transition, EMT）有关。Yoshida等^[36]^的研究，通过评价共培养*EGFR*突变的肺腺癌细胞系和podoplanin-posiotive CAFs对EGFR-TKIs吉非替尼治疗的敏感性，探讨EGFR基因突变的NSCLC对EGFR-TKI靶向治疗的敏感性是否受podoplanin-positive CAFs的影响。其结果和对照组相比，共培养了podoplanin-positive CAFs肺腺癌细胞系表现出更强的耐药性，对于术后复发的患者，podoplanin-positive CAFs组和podoplanin-negative CAFs组相比，总体表现出对EGFR-TKIs更低的治疗反应（53% *vs* 83%; *P* < 0.01）。可见，podoplanin-positive CAFs对EGFR-TKIs原发性耐药起重要作用，是一个理想的治疗靶点，可联合EGFR-TKIs用于治疗*EGFR*突变的NSCLC患者。值得注意的是，Ishibashi等^[37]^探讨了CD200-positive CAFs对*EGFR*突变的肺腺癌PC9细胞系EGFR-TKIs吉非替尼治疗的敏感性的影响。结果显示，当共培养PC9和CD200-positive CAFs，可以增强吉非替尼对肿瘤细胞的杀伤效应，而对于未加入吉非替尼培养基组内，肿瘤细胞的生存不受影响。同时，他们也对术后复发的肺腺癌患者手术切除的样本进行免疫组化的分析，含有CD200-positive CAFs患者组，其接受吉非替尼治疗后的无进展生存期（progression-free survival, PFS）明显延长。因此他们认为，CD200-positive CAFs可以增强EGFR-TKIs治疗的敏感性，拥有更深远的临床应用价值。综上所述，CAFs可影响*EGFR*突变的肺腺癌细胞系对EGFR-TKIs分子靶向治疗的敏感性，其机制可能和CAFs诱导EMT现象和激活Hh信号通路有关，但仍需要进一步研究证实。而在EGFR-TKIs耐药的NSCLC患者中，针对CAFs靶点研究值得进一步深入，其有望与EGFR-TKIs联合治疗，进一步延长肿瘤患者PFS。

### 1.5 髓源性抑制细胞（myeloid-derived suppressor cells, MDSC）

MDSC是一类具有免疫抑制功能的细胞群，参与抗肿瘤免疫抑制，促进肿瘤细胞发生免疫逃逸^[38]^。Holdman等^[39]^在乳腺癌复发机制的研究中，对荷瘤小鼠使用成纤维细胞生长因子受体（fibroblast growth factor receptor, FGFR）抑制剂BGJ398后，发现肿瘤快速消退伴有MDSC水平显著降低，在停止治疗后，大多在1个月-4个月出现肿瘤复发。复发的肿瘤组织内发现磷酸化的EGFR表达和MDSC水平升高，考虑EGFR信号通路激活参与了肿瘤的复发。而联合使用EGFR抑制剂拉帕替尼组与单用BGJ398组相比，肿瘤复发时间延长，MDSC水平再次降低。结论为EGFR信号上调和乳腺癌复发有关，联合EGFR抑制剂治疗可以延长PFS。研究者认为，此项研究虽然没有直接阐明MDSC水平是否能影响EGFR-TKI疗效，但间接表达MDSC水平与EGFR信号、肿瘤复发之间存在着一定的联系。Feng等^[40]^研究认为，S100A9 MDSC是EGFR突变的晚期肺腺癌患者使用吉非替尼一线治疗PFS的独立危险因素之一（HR=4.944, 95%CI: 1.578-15.490, *P*=0.006），与低水平MDSC患者相比，高水平MDSC患者的PFS更短[中位PFS 7.2个月（95%CI: 5.5-8.9）*vs* 12.7个月（95%CI: 8.1-17.2），*P*=0.014]。可见S100A9 MDSC可能影响EGFR-TKIs的治疗反应。

## 2 PD-1及其配体PD-1L/PD-2L与EGFR-TKIs疗效的关系

PD-1是一种重要的免疫抑制分子，在人类和小鼠的多种细胞（T细胞、B细胞、NK细胞、DC以及活化的单核细胞等）均有表达，而其配体PD-1L/PD-2L常表达于肿瘤细胞表面，包括NSCLC。并且，大约有20%的晚期NSCLC患者受益于PD-1阻断剂，第一代PD-1阻断剂pembrolizumab和nivolumab于2015年获得美国食品药品监督管理局（Food and Drug Administration, FDA）批准用于晚期NSCLC患者的二线治疗^[41]^。值得注意的是，在基因突变的NSCLC患者中，EGFR信号可以上调PD-1、PD-1L的表达，进而调节免疫逃逸反应，影响EGFR-TKIs疗效^[42, 43]^，其机制可能和IL-6/Janus激酶/信号转导和转录激活因子3（IL-6/JAK/STAT3）信号通路有关^[44]^。Lin等^[45]^的临床队列研究中，通过免疫组织化学法检测接受EGFR-TKIs的晚期NSCLC肿瘤组织内的PD-1和PD-1L表达，多变量分析模型和生存分析模型验证PD-1/PD-1L的表达与患者PFS和总生存期（overall survival, OS）的关系。有53.6%的患者阳性表达PD-1L，表明在这些患者中，至少有一半的患者可以联合接受PD-1L阻滞剂治疗，并可能从中获益。结果显示，和不表达PD-1L的NSCLC患者相比，阳性表达PD-1L拥有更长的PFS（HR=0.46, *P*=0.014）和OS（HR=0.26, *P*=0.002）；而阳性表达PD-1与不表达PD-1的NSCLC患者相比，PFS（*P*=0.907）和OS（*P*=0.640）无明显差异。因此认为PD-1L或许可以作为NSCLC患者*EGFR*基因突变和接受EGFR-TKIs抑制剂临床预后的生物学标志物。PD-1/PD-1L抑制剂的成功问世，是晚期EGFR-TKIs耐药的NSCLC患者的福音，目前推荐单用二线治疗。对EGFR-TKIs获得性耐药患者，联合使用PD-1/PD-1L抑制剂和EGFR-TKIs似乎是更有前景的治疗策略，而近期的系列临床实验结果显示：在化疗前或化疗后的NSCLC，联合使用osimertinib和durvalumab显示了更优的临床结局，但这种联合提高了间质性肺病的发生率（38%）；吉非替尼联合durvalumab的治疗作用同样显著，但3级-4级肝毒性的发生率显著升高（40%-70%）；同样几组联合方式，但均显示3级-4级不良事件的增加；总的来说，对NSCLC联合使用PD-1/PD-1L抑制剂和EGFR-TKIs，因其治疗相关的毒性反应发生率高而受到限制，但仍然不失为一个有前景的治疗策略，有待进一步研究^[46]^。

## 3 总结

肿瘤微环境是肿瘤细胞赖以生存和发展的复杂环境，而这微环境内的免疫细胞及其调节方式对肿瘤的发生和发展起着重要的作用。如今，分子靶向治疗开启了肿瘤治疗的新潮流，而如何应对分子靶向药物耐药或者疗效不佳成为了我们新的挑战。本篇综述从肿瘤微环境免疫细胞及其调节方式以及PD-1/PD-1L的角度出发，阐述了其可能对EGFR-TKIs疗效的影响，为免疫治疗和分子靶向治疗提供新的契合点。
